# Myosteatosis: an emerging biomarker of aging

**DOI:** 10.3389/fragi.2026.1868010

**Published:** 2026-09-03

**Authors:** Lauren S. Roe, Joseph M. Zmuda, Iva Miljkovic

**Affiliations:** 1 Division of Geriatrics, Department of Medicine, University of Pittsburgh, Pittsburgh, PA, United States; 2 Department of Epidemiology, University of Pittsburgh School of Public Health, Pittsburgh, PA, United States

**Keywords:** computed tomography, epidemiology, intermuscular adipose tissue, intramuscular adipose tissue, intramyocellular lipids, longevity, magnetic resonance imaging, magnetic resonance spectroscopy

## Abstract

Adipose tissue infiltration into skeletal muscle (i.e., myosteatosis) has emerged as an independent contributor to metabolic disorders and declines in skeletal muscle function with age. It remains unclear whether myosteatosis is a marker of aging or simply a consequence of disease or disuse. This review summarizes evidence from population-based epidemiologic studies that utilized non-invasive imaging to measure myosteatosis and to evaluate age-related changes among community-dwelling middle aged and older adults. Myosteatosis consistently increased with age, independent of weight change, and the burden of myosteatosis was greater among women, and non-White racial and Hispanic ethnic groups after accounting for physical activity levels, chronic disease burden, and overall body size. Myosteatosis accumulation is not uniform throughout the body and is differentially associated with clinically meaningful age-related outcomes, including changes to gait, balance, muscle strength, and physical function. Hormonal, cellular, genetic, and lifestyle differences comprise the biological pathways that may lead to age-related increases in myosteatosis. Future work in diverse populations using standardized imaging methodologies could include longitudinal measures of biological markers to better understand the biological precursors that accompany the phenotypic change. In conclusion, evidence from several well-characterized epidemiologic studies is consistent with myosteatosis an independent marker of aging.

## 1 Age-related changes in body composition and skeletal muscle

The older adult population is increasing rapidly and the number of older adults aged ≥65 years recently outnumbered children aged <5 years United Nations, Department of Economic and Social Affairs, Population Division (2024), meaning the number of people with chronic conditions is expected to increase. With age, several physiological changes occur at all levels and in all systems, contributing to the development of disease and disability. The most visibly noticeable changes occur in body composition and are broadly characterized by muscle mass and bone mineral density decreases, and fat mass increases, particularly fat mass accumulating in non-adipose tissue and organs (i.e., ectopic fat). Some of the hallmarks of body composition changes with age include changes to skeletal muscle function, composition, and metabolism, signifying a critical area of study to target healthy aging.

**FIGURE 1:**
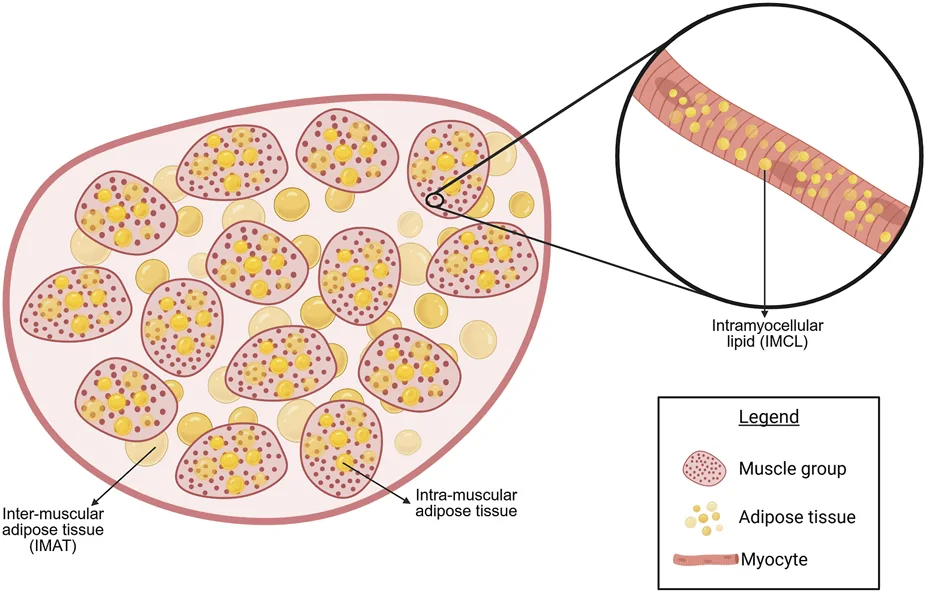
Representation of ectopic fat depots in skeletal muscle (myosteatosis). Created in BioRender. Roe (2026) https://BioRender.com/q6tp5d2.

Ectopic fat infiltration into the skeletal muscle is also known as myosteatosis. There are three main depots that comprise myosteatosis (Figure 1): a) inter-muscular adipose tissue (IMAT) – the visible extracellular adipose tissue between muscle groups that gives muscle a “marbled” appearance; b) intra-muscular adipose tissue–extracellular adipose tissue within the individual muscle, characterized as muscle density or muscle attenuation from imaging methods; and c) intramyocellular lipids (IMCL) – lipids within individual myocytes. Myosteatosis may play a critical role in development of major age-related conditions like metabolic dysfunction (Carobbio et al., 2011; Goodpaster et al., 2023), mobility impairment (Santanasto et al., 2019; Visser et al., 2005), cognitive impairment (Acevedo-Fontánez et al., 2023), and survival (Larsen et al., 2020; Miljkovic et al., 2015; Reinders et al., 2016; Zhao et al., 2016), but is still largely understudied compared to other ectopic fat depots, like visceral adipose tissue. In particular, less is known about age-related changes in myosteatosis and its association with age-related physical and metabolic decline, and it remains unclear if myosteatosis is a marker of aging (Zhang et al., 2025), a consequence of disuse, or a consequence existing or developing disease (Addison et al., 2014b).

**FIGURE 2:**
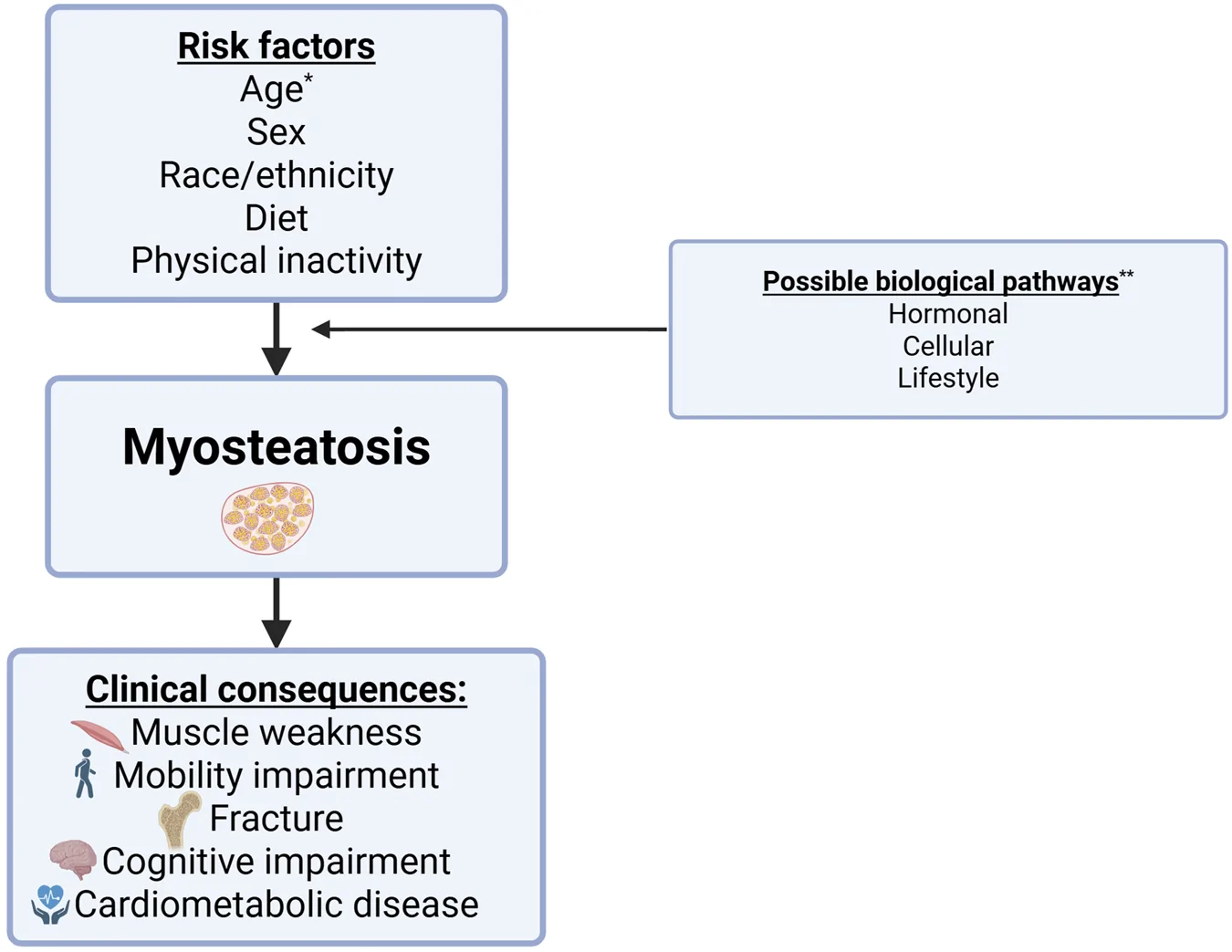
Conceptual framework of myosteatosis as a marker of aging, its potential biological pathways, and its association with poor clinical outcomes. ^*^Primary focus of this review. ^**^Biological pathways leading to increased myosteatosis are not fully understood and still under investigation, so this is not a comprehensive list. Created in BioRender. Roe, L. (2026) https://BioRender.com/0u0itvp.

There is currently no gold standard measurement method to quantify myosteatosis (Correa-de-Araujo et al., 2020; Correa-de-Araujo et al., 2017). Non-invasive imaging like computed tomography (CT), magnetic resonance imaging (MRI), and magnetic resonance spectroscopy (MRS) are commonly used in large-scale population-based research studies because they are widely available. CT typically uses radiation attenuation (measured in Hounsfield units [HU]) to distinguish skeletal muscle from adipose tissue and to measure IMAT and intra-muscular adipose tissue (Aubrey et al., 2014). MRI can segment muscle compartments to determine intra-muscular adipose tissue and IMAT. Finally, MRS is a specialized imaging technique from MRI that can quantify IMCL. This review will summarize the existing epidemiologic literature on image-derived myosteatosis changes with age, with a particular focus on accelerated changes in adults and older adults. Upon this background, we review literature on the change in myosteatosis with age from epidemiologic studies of the general community-dwelling population and summarize possible mechanisms for these changes. Specifically, we hypothesize that epidemiologic evidence supports myosteatosis as a marker of aging rather than simply a consequence of disease or disuse (Figure 2), and that it also plays a role in poor health outcomes in older adults.

## 2 Myosteatosis and aging among the general population of older adults

The first epidemiologic studies of myosteatosis in humans were published around the year 2000 with the aim of understanding if muscle adipose tissue infiltration was a determinant of muscle strength in a cohort of community-dwelling older adults (Goodpaster et al., 2001). Age-related muscle strength declines (∼4%/year) outpaced dual-energy x-ray absorptiometry (DXA)-measured lean mass declines (∼1%/year) (Goodpaster et al., 2006), suggesting that physiological changes other than lean mass contribute to loss of strength. Recent studies using more accurate measures of muscle mass from D_3_-creatine dilution methods in epidemiologic studies show a stronger association between muscle mass, physical performance, and strength than previously appreciated (Hetherington-Rauth et al., 2025; Orwoll et al., 2022), meaning muscle mass likely plays a stronger role in function than previously thought. Nonetheless, these recent studies continue to highlight myosteatosis as a strong, independent predictor of muscle performance that needs to be studied further (Orwoll et al., 2022).

Myosteatosis impacts pathways leading to metabolic dysfunction, so there has been particular interest in aging research to understand the role of myosteatosis in muscle function decline and chronic disease development. However, older adults tend to be physically inactive and have many chronic conditions, making it challenging to understand the determinants of myosteatosis (aging or disease/disuse) and thus, its role in muscle function declines. Several large-scale, population-based studies describe cross-sectional differences (Table 1) and longitudinal changes across adulthood (Table 4) to understand the evolution of myosteatosis across the lifespan.

**TABLE 1:** Cross-sectional epidemiologic cohort studies using image-derived measures of myosteatosis by age in the general population of adults and older adults.

References	Cohort	Sample size	Age (yrs)	Image method	Myosteatosis depot (unit)	Anatomical location	Analysis	Covariates	Result
Machann et al. (2005)	TUebinger Lifestyle Intervention Program	150	19–69	^1^H-MRS	IMCL (% of water signal)	Tibialis anterior, soleus	Correlation by sex	Unadjusted	↔ Men: r = 0.0–0.01 Women: r = −0.09-0.18
Miljkovic-Gacic et al. (2008a)	Tobago Health Study (men only)	1,249	40–91	pQCT	IMAT (mm^2^)	Calf	ANCOVA across 10-year age group	Height, total adipose tissue, total muscle area	↑ with age (p-trend<0.001)
Miljkovic et al. (2013)	MrOS (men only)	393	≥65	QCT	IMAT (cm^3^)	Abdomen	ANCOVA across 5-year age group	Study site, height, muscle volume, total body fat percent	Abdominal muscle: ↑ with 5-year age group, p-trend = 0.003 Paraspinal muscle: ↑ with 5-year age group, p-trend<0.001 Psoas muscle: ↔ between 5-year age group, p-trend = 0.09
Dai et al. (2023)	United Kingdom Biobank	13,062	45–74	MRI	Muscle fat infiltration (%)	Neck to knee	T-test within sex by age group	Unadjusted	↑ in 60–74 years versus 45–59 years in men and women (both p < 0.001)
Anderson et al. (2025)	United Kingdom Biobank	50,332	45–84	MRI	IMAT (%)	Thigh	Means by 10-year age group	Unadjusted	↑ with 10-year age group in men and women (no p-value evaluated)

Abbreviations: ↔ = no difference; ↑ = increase or higher; ↓ = decrease or lower; IMCL = intramyocellular lipids; ^1^H-MRS = proton magnetic resonance spectroscopy; IMAT = intermuscular fat; QCT = quantitative computed tomography; pQCT = peripheral quantitative computed tomography; ANCOVA = analysis of covariance.

Miljkovic et al. (2008b) were one of the first to describe cross-sectional differences in myosteatosis by age group using CT-measured calf IMAT. Among African Caribbean men aged ≥40 years, IMAT was significantly higher with every 10-year increase in age after considering body size and muscle cross-sectional area (Table 1) (Miljkovic-Gacic et al., 2008a). Similar cross-sectional results from The Osteoporotic Fractures in Men (MrOS) Study showed that IMAT was significantly higher with each 5-year age group in the abdominal and paraspinal muscles (Table 1) (Miljkovic et al., 2013). Conversely, cross-sectional findings of IMCL measured from MRS showed no significant correlation with age at the tibialis anterior (men: r = 0.0, women: r = −0.09) or the soleus (men: r = −0.01, women r = 0.18; Table 1) (Machann et al., 2005). However, results were unadjusted, conducted in a smaller sample across adulthood (n = 150, age range: 19–65 years), and evaluated a different muscle depot than the Tobago Health Study and MrOS, so results are not directly comparable.

Like IMAT, age-related declines in muscle density, a surrogate measure of intra-muscular adipose tissue, have been reported. Goodpaster et al. (2001) showed a decline in thigh muscle density (e.g., increased intra-muscular adipose tissue) among men and women aged ≥70 years in the Health, Aging and Body Composition (Health ABC) study (p-value not reported; Table 3) (Goodpaster et al., 2001). Lauretani et al. (2006) later found that calf muscle density significantly declined with age among adults aged 21–96 years, with a steeper decline in density starting at age 65 years in men and 60 years in women (Table 2) (Lauretani et al., 2006). Wu et al. (2024) found similar age-related declines in abdominal muscle density in Chinese adults aged 21–80 years and observed steeper declines starting at age 55 in men and 45 in women (Wu et al., 2024). Trunk muscle density was higher among younger (25–50 years) versus older (75–87 years) adults in the Framingham Heart Study (FHS) Offspring cohort, after adjusting for body size, muscle size, and self-reported physical activity (Table 2) (Anderson et al., 2013). Among adults and older adults in the FHS Offspring and Third Generation cohorts, trunk muscle density also significantly decreased with every 10-year age group (Table 2) (Johannesdottir et al., 2018). Similarly, thigh and abdominal muscle density were significantly lower with every 5-year age group after adjusting for body size and muscle size among older men and women in the Age, Gene/Environment Susceptibility (AGES)-Reykjavik Study (Table 2) (Figueiredo et al., 2021).

**TABLE 2:** Cross-sectional epidemiologic studies that compared image-derived measures of myosteatosis between sex in the general population of adults and older adults.

References	Cohort	Sample size	Age (yrs)	Image method	Myosteatosis depot (unit)	Anatomical location	Analysis	Covariates	Result
Lauretani et al. (2006)	InCHIANTI	1,162	21–96	pQCT	Muscle density (mg/cm^3^)	Calf	• Age: linear regression by sex • Between sex: not described	Unadjusted	Age: ↓ with age; steeper decline starting at age 65 years (men) and 60 years (women) Sex: ↑ in men vs. women (p < 0.001)
Anderson et al. (2013)	Framingham Heart Study (FHS) Offspring	120	25–50, 75–87	CT	Muscle density (HU)	Trunk	General linear mixed model	Muscle cross-sectional area, height, BMI, self-reported physical activity level (Physical Activity Index) to estimate total energy expenditure	Age group: ↓ with age (all p < 0.05) Sex: ↑ in men vs. women (p < 0.05)
Johannesdottir et al. (2018)	FHS Offspring and Third Generation	250	40–90	CT	Muscle density (HU)	Trunk	By age and sex: general linear mixed model	Height and weight	10-year age group: ↓ with 10-year age group (p < 0.05) Sex: ↑ in men versus women (p < 0.05)
Figueiredo et al. (2021)	Age, Gene/Environment Susceptibility- Reykjavik Study	5,331	66–96	CT	Muscle density (HU)	Abdomen	• Between sex: t-test • ANOVA • 5-year age group: linear mixed model	BMI, education, self-reported MVPA^*^, health status, smoking, health conditions, alcohol, hs-CRP, muscle area, intramuscular adipose tissue area	Age group: ↓ with 5-year age group (p < 0.001) Sex: ↑ in men versus women (p < 0.001)
Wu et al. (2024)	China Action on Spine and Hip	4,120	21–80	CT	Muscle density (HU)	3rd and 5th lumbar vertebrae	T-test or Mann-Whitney rank test	Unadjusted	Age: ↓ with 5-year age group Sex: ↑ in men versus women (p < 0.001)

Abbreviations: ↑ = increase or higher; ↓ = decrease or lower; CT, computed tomography; pQCT, peripheral quantitative computed tomography; HU, houndsfield units; ANOVA, analysis of variance; hs-CRP, high sensitivity C-reactive protein.

* MVPA, collected using the following question “During the last 12 months how often did you participate in moderate to vigorous activities (MVPA)”, with responses as (1) never, (2) rarely, (3) weekly but less than 1 h per week, (4) 1–3 h per week, (5) 4–7 h per week, or (6) more than 7 h per week (Figueiredo et al., 2021).

More recently, cross-sectional findings further support an increase in myosteatosis with age among a large sample from the UK Biobank (n = 13,062; Table 1) (Dai et al., 2023). Dai et al. (2023) established population norms for MRI-measured muscle adipose tissue infiltration for healthy men and women aged 45–74 years and found that whole body muscle adipose tissue infiltration (from neck to the knee) was significantly higher in older adults. However, “muscle fat infiltration” was not defined, so we cannot determine which myosteatosis depot is driving the age-related difference. The results presented also did not adjust for physical activity or chronic conditions, though authors noted that they excluded anyone with conditions, medications, or surgeries that would impact fat mass distribution. Additionally, authors only included adults of European ancestry up to age 74 years, so results are not generalizable to other racial or ethnic groups or older adults ≥75 years. Two years later, Anderson et al. (2025) published cross-sectional data from a larger UK Biobank sample (n = 50,332) that included adults aged 45–84 years and similarly found that IMAT increased with every 10-year age group, but they did not perform any statistical tests to determine if this was a significant trend (Table 1) (Anderson et al., 2025).

Thus, there is consistent evidence from cross-sectional studies in several well-characterized cohorts of older adults (e.g., Tobago Health Study, Health ABC, MrOS, FHS, AGES-Reykjavik, UK Biobank) showing age-related increases in IMAT and intra-muscular adipose tissue in upper and lower body and trunk muscle groups. The consistency of findings across large samples provides robust evidence of age-related increases in myosteatosis from cross-sectional studies. While the cross-sectional design limits our understanding of within person changes in myosteatosis with age, there are some studies that report longitudinal changes with age. The longitudinal studies are critical for informing age-related changes in myosteatosis, as they inherently account for within person variability over time instead of relying on differences across age groups that may result from lifestyle, generational, or health status differences across age groups. Evidence provided from these longitudinal studies is stronger than that of cross-sectional studies, particularly when follow-up time is extensive to understand the natural history of changes over time. While the published data on longitudinal studies is few (Table 4), the evidence is robust and more strongly supports the age-related increase in myosteatosis.

Seminal findings from Health ABC suggested for the first time that myosteatosis is a marker of aging. Authors reported significant longitudinal increases in thigh IMAT among Black and White healthy and high-functioning men and women aged ≥70 years, even among those who lost weight during the follow-up period (Table 4) (Delmonico et al., 2009). Miljkovic et al. (2016) similarly reported a significant 6-year increase in calf IMAT and a decrease in muscle density after adjusting for change in body weight in African Caribbean men aged ≥40 years (Table 4) (Miljkovic et al., 2016). A progressive trend across age groups was statistically significant for muscle density (40–54 years: 2.9% ± 3.7%; 55–64 years: 3.1% ± 3.9%; 65+ years: 4.7% ± 5.9%; p-trend<0.001) suggesting for the first time that intra-muscular adipose tissue accumulation accelerates with increasing age among middle aged and older men (Miljkovic et al., 2016). Authors did not observe a significant trend across age group for IMAT and concluded that this could be due to survival bias because IMAT (not muscle density) is associated with an increased risk of type 2 diabetes in this population; those with higher IMAT at baseline may not have been able to participate in the follow-up visit due to poorer health. Another smaller sample from the Osteoarthritis Initiative showed that MRI-measured thigh IMAT significantly increased over 2 years, regardless of presence of radiographic osteoarthritis (Table 4) (Beattie et al., 2012). In the longest follow-up study to date, IMAT significantly increased over 18 years among middle age and older adults in the Tobago Health Study, regardless of baseline age (Roe et al., 2025). Moreover, increases in myosteatosis preceded changes in muscle area and were more strongly associated with muscle strength (Roe et al., 2025).

The evidence presented from cross-sectional, but more importantly, longitudinal results further support an age-related increase in IMAT and decrease in muscle density in many muscle groups among the general adult population, independent of weight change. While there is strong longitudinal evidence of an increase in myosteatosis with age, there are few published studies in diverse populations in both men and women, highlighting an important gap in the literature. Additionally, future studies should distinguish if weight change over time was intentional or unintentional, as each has different physiological implications (Wannamethee et al., 2005). Physical activity and disease status were not adjusted for in these studies, so further studies are needed to tease apart age vs. disease/disuse as a determinant of increased myosteatosis in the general population of adults. Other studies have compared myosteatosis by sex and/or race, while adjusting for lifestyle factors and disease. Given that age-related changes in myosteatosis may depend on several other factors, such as sex, race/ethnicity, lifestyle (e.g., aerobic fitness levels, physical activity, etc.), as well as anatomical region, below we present existing evidence that can help further clarify the age-related changes in myosteatosis.

## 3 Importance of differences in myosteatosis with age by sex, race, and anatomical region

### 3.1 Importance of sex

Adipose tissue distribution with age varies by sex, where women often store adipose tissue in the lower body and men store adipose tissue in visceral depots (Varlamov et al., 2014). Hormonal differences by sex lead to dramatic differences in age-related changes in other tissues like bone (Drake et al., 2015), which could suggest that myosteatosis storage with age differs by sex as well. Much of the literature that statistically compares myosteatosis across sex in the general population of older men and women focuses on muscle density (e.g., intra-muscular adipose tissue), with only two analyses in Health ABC evaluating IMAT by sex (Tables 3, 4) (Delmonico et al., 2009; Goodpaster et al., 2001). Other studies that evaluate myosteatosis by sex focus on differences across racial groups within sex (vs. across sex) (Eastwood et al., 2014; Shah et al., 2016) (Table 3), which will be discussed below (see “Importance of race/ethnicity”).

**TABLE 3:** Cross-sectional epidemiologic cohort studies that compared image-derived measures of myosteatosis by race in the general population of adults and older adults.

References	Cohort	Sample size	Age (yrs)	Image method	Myosteatosis depot (unit)	Anatomical location	Analysis	Covariates	Result
Goodpaster et al. (2001)	Health ABC	2,627	70–79	CT	IMAT (cm^2^), muscle density (HU)	Thigh	ANOVA	Unadjusted	IMAT: ↔ between men vs. women (p = 0.08) ↑ in Black vs. White men (p < 0.01) ↑ in Black vs. White women (p < 0.01) Muscle density: ↓ with age (p-value not provided) ↑ between men vs. women (p < 0.01) ↓ in Black vs. White women (p < 0.01) ↔ between Black vs. White men (p = 0.17)
Gallagher et al. (2005)	Studies at St. Luke’s Roosevelt Hospital’s Body Composition Unit	338	≥18	MRI	IMAT (kg)	Whole body	Generalized linear model	Age, weight squared, total adipose tissue, sex race by total adipose tissue interaction	↑ in African American vs. White men and women at higher levels of total adiposity (p < 0.05)
Delmonico et al. (2009)	Health ABC	1,678	70–79	CT	IMAT (cm^2^), muscle density (HU)	Thigh	T-test	Unadjusted	IMAT: ↑ in Black vs. White men (p < 0.001) ↑ in Black vs. White women (p < 0.001) Muscle density: ↔ between Black vs. White men (p > 0.05) ↓ in Black vs. White women (p < 0.001)
Miljkovic et al. (2009a)	Tobago Health Study and MrOS (both men only)	Tobago Health Study: 518 MrOS: 1,105	Tobago Health Study: 72 MrOS: 77	pQCT	IMAT (mm^2^)	Calf	Generalized linear model	Age, height, calf skeletal muscle area, total calf adipose tissue, self-reported hours/wk walking, smoking, alcohol	↑ in African American vs. White (p < 0.001)
Eastwood et al. (2014)	SABRE	1,183	56–85	CT	IMAT (cm^2^), muscle density (cm^2^)	Thigh	ANCOVA	Age and thigh muscle area, or age, height, and fat mass	IMAT: ↑ European vs. South Asian men (p < 0.001) and women (p = 0.02), adjusting for age and thigh muscle area Muscle density: ↑ in European vs. South Asian men (p < 0.001) and women (p = 0.03), adjusting for age and thigh muscle area
Shah et al. (2016)	MASALA and MESA	MASALA: 906 MESA: 1,944	MASALA: 40–84 MESA: 1,944	CT	IMAT (cm^2^)	Abdomen	Generalized linear model	Age, sex, study site, alcohol use, smoking history, education, household income, exercise, BMI, hypertension, diabetes, high-density lipoprotein, triglycerides	↑ in South Asians vs. all other race/ethnic groups (all p < 0.01)
Tejani et al. (2025)	United Kingdom Biobank	38,824 White Europea, 397 South Asians	40–69	MRI	Muscle fat infiltration (%)	Thigh	Multiple linear regression	Sex, age, height, weight, type 2 diabetes, cardiovascular disease, self-reported physical activity level (IPAQ; moderate, low, high), smoking, alcohol, employment status, education level, Townsend deprivation index	↑ in South Asians vs. White European (p < 0.001)

Abbreviations: ↔ = no difference; ↑ = increase or higher; ↓ = decrease or lower; IMAT, intermuscular fat; CT, computed tomography; pQCT, peripheral quantitative computed tomography; MRI, magnetic resonance imaging; HU, hounsfield units; ANOVA, analysis of variance; ANCOVA, analysis of covariance; MESA, Multi-Ethnic Study of Atherosclerosis; MASALA, Mediators of Atherosclerosis of South Asians Living in America.

**TABLE 4:** Longitudinal epidemiologic cohort studies using image-derived measures of myosteatosis by age in the general population of adults and older adults.

References	Cohort	Follow-up (yrs)	Sample size	Age (yrs)	Image method	Myosteatosis depot (unit)	Anatomical location	Analysis	Covariates	Result
Delmonico et al. (2009)	Health ABC	5	1,678	70–79	CT	IMAT (cm^2^)	Thigh	Paired t-test	Unadjusted	↑ (p < 0.001 for men and women) ↔ between sex, within race (p-value not provided)
Beattie et al. (2012)	Osteoarthritis Initiative (women only)	2	86	45–79	MRI	IMAT (cm^3^)	Thigh	Paired t-test, ANCOVA	Age	↑ (unadjusted: p < 0.05; age-adjusted: p > 0.05)
Miljkovic et al. (2016)	Tobago Health Study (men only)	6	1,515	≥40	pQCT	IMAT (cm^2^), muscle density (mg/cm^3^)	Calf	• Paired t-test for 6-year Δ • ANCOVA (Δ by 15-year age group and Δ by weight change)	Follow-up time, baseline IMAT/muscle density, muscle area, type 2 diabetes	% Δ in IMAT: ↑ over 6 years (overall and by weight Δ group: p < 0.001) ↔ between 15-year age group (p-trend = 0.22) % Δ in muscle density: ↓ over 6 years (overall and by weight Δ group: p < 0.001) ↑ with 15-year age group (p-trend<0.001)
Roe et al. (2025)	Tobago Health Study (men only)	18	339	≥40	pQCT	IMAT (cm^2^)	Calf	GEE	Baseline age	↑ (p < 0.001)

Abbreviations: ∆ = change; ↔ = no difference; ↑ = increase or higher; ↓ = decrease or lower; IMAT, intermuscular fat; QCT, quantitative computed tomography; pQCT, peripheral quantitative computed tomography; ANOVA, analysis of variance; ANCOVA, analysis of covariance; GEE, generalized estimating equation.

The Health ABC studies that compared IMAT across sex evaluated cross-sectional (Goodpaster et al., 2001) and longitudinal (Delmonico et al., 2009) differences. Cross-sectionally, Goodpaster et al. (2001) found that thigh IMAT did not significantly differ between 1,285 men (10.0 ± 7.2 cm^2^) and 1,342 women (10.4 ± 6.0 cm^2^; p = 0.08) (Goodpaster et al., 2001). Delmonico et al. (2009) found that men and women both experienced a significant 5-year increase in IMAT, but the relative magnitude of change was greater in men (48.5% ± 59.6%; p < 0.001) compared to women (29.0% ± 43.6%; p < 0.001) (Delmonico et al., 2009). Importantly, this pattern was observed in men and women who were weight stable, lost weight, or gained weight, suggesting that this sex difference occurs regardless of weight change over time, though intentionality of weight loss was not specified. Thus, older men and women may initially have similar amounts of IMAT, but men gain more IMAT over time compared to women. Given results are from a single cohort, more longitudinal evidence from epidemiologic studies is needed to support these findings in other cohorts of middle-aged and older adults.

Evidence of sex differences in muscle density is more abundant and patterns are more clear from several epidemiologic studies, including InCHIANTI (Lauretani et al., 2006), Health ABC (Goodpaster et al., 2001), FHS Offspring and Third Generation cohorts (Anderson et al., 2013; Johannesdottir et al., 2018), AGES-Reykjavik Study (Figueiredo et al., 2021), and the China Action on Spine and Hip (CASH) cohort (Wu et al., 2024). The results from these cohorts consistently found that older men had significantly greater muscle density compared to older women in the calf, trunk, abdominal, and thigh muscles. In Health ABC, Goodpaster et al. (2001) found that men had significantly greater thigh muscle density compared to women (men: 37.3 ± 6.5 HU; women: 35.5 ± 7.0 HU; p < 0.001) (Goodpaster et al., 2001). Interestingly, this contrasts the null association by sex for IMAT mentioned earlier in this sample. However, no other epidemiologic studies in older adults evaluated both IMAT and muscle density to further understand sex-related differences.

Results from the InCHIANTI cohort in Tuscany, Italy (n = 1,162) reported that men had significantly higher muscle density compared to women, but results were completely unadjusted (Table 2) (Lauretani et al., 2006). Data from the FHS Offspring study similarly found that men compared women to had 5.0–14.7 HU greater muscle density in all trunk muscles (all p < 0.05) except for the external oblique and the psoas major (p > 0.05), regardless of body mass index and self-reported physical activity (Table 2) (Anderson et al., 2013). Interestingly, there was no significant interaction between age (range: 35–87 years) and sex, suggesting that though men had higher muscle density, the magnitude of difference by sex did not vary by age (Anderson et al., 2013). This is in contrast with longitudinal results by sex from Health ABC where there was no cross-sectional difference in IMAT (Goodpaster et al., 2001), but the relative change in IMAT was greater over time in men compared to women (Delmonico et al., 2009). However, this notion of equal magnitude of change in muscle density from the FHS Offspring analysis should be interpreted with caution, as the analysis was conducted using cross-sectional data.

Another FHS study in the Offspring and Third Generation cohorts (n = 250 adults and older adults) support these findings by similarly reporting that men had 5%–68% greater trunk muscle density compared to women (depending on the muscle), after adjusting for height, weight, and age (Table 2) (Johannesdottir et al., 2018). Self-reported physical activity index score also did not vary greatly by age or sex, suggesting that sex differences were likely not due to differences in physical activity. Among Chinese adults in the CASH cohort, abdominal muscle density was significantly higher in men compared to women, but results were completely unadjusted (Wu et al., 2024). Finally, among older adults in the AGES-Reykjavik Study, the pooled muscle density for the thigh and abdominal muscles was significantly higher for men (40.5 ± 6.8 HU) compared to women (37.4 ± 7.7 HU; p < 0.001). For this population in particular, the difference by sex may be because of differences in health and behavior characteristics, as men tended to be more educated and physically active, but reported fewer health conditions and poor health status compared to women.

These results of higher myosteatosis in women compared to men track with observed differences in muscle function by sex among older adults. Around age 15 years, females have about ∼75% of grip strength as that of males (Nuzzo, 2023). Grip strength peaks for both sexes in mid-life, but the magnitude of increase from adolescence to middle age is greater for males than females and this difference persists throughout adulthood into older adulthood (Nuzzo, 2023). The difference in strength likely occurs because men tend to have greater type II muscle fiber area compared to women (Nuzzo, 2023; James et al., 2025), which also tracks with men having less muscle adipose tissue infiltration compared to women since type II fibers contain less adipose tissue. On a hormonal level, there are differences in leptin, estrogen, and testosterone that may contribute to sex differences. Older women tend to have lower leptin levels compared to older men (Isidori et al., 2000), which may lead to greater muscle adipose tissue infiltration in women. Estrogen and testosterone levels decline with age (Cheng et al., 2024). Androgens are converted to estrogens within adipose tissue. Estrogen has several positive health effects, including muscle repair and regeneration, anti-inflammatory properties (Enns and Tiidus, 2010), and reduces adipose tissue accumulation (Cooke and Naaz, 2004). Accordingly, the declining levels of estrogen with age could increase myosteatosis. Similarly, declining testosterone with age can increase adipose tissue storage into the muscles in both men (Woodhouse et al., 2004) and women (Ofori et al., 2019) by impacting protein synthesis and metabolism.

Taken together, it appears that there may be a sex difference in IMAT among older adults. However, results for IMAT come from a single study, so other epidemiologic cohorts in middle-age and older adults need to compare IMAT by sex in the general population of older adults to confirm results. The collective results of intra-muscular adipose tissue as measured by muscle density, however, are clear and persist regardless of differences in body size and physical activity: men have higher muscle density compared to women. However, each of the studies on muscle density were cross-sectional, so the within person longitudinal changes by sex are unclear. Additionally, many studies did not account for disease status, so it is unclear if these sex differences are due to differences in disease prevalence by men and women. Finally, few studies have evaluated associations between muscle adipose tissue and biological changes to determine the drivers of sex differences with age.

### 3.2 Importance of race and ethnicity

Compared to White adults, the burden of obesity and diabetes is greater among Native American, non-Hispanic Black and people of African ancestry, non-Hispanic Asian, and Hispanic adults (U.S., 2023; Cheng et al., 2019; Deng et al., 2025; Taylor et al., 2010). These differences cannot be explained by differences in traditional risk factors like body fat, waist circumference, or visceral or subcutaneous adipose tissue distribution (Liu et al., 2010). Moreover, people of African ancestry are at greater risk of developing additional chronic conditions like poor physical function, as they also tend to have slower gait speed than White adults (White et al., 2013). Given these racial differences in metabolism and function, studies have compared myosteatosis by race with age to understand if muscle adiposity is the culprit for this disproportionate risk. In this section, we present available findings of age-related changes in myosteatosis by race from well-characterized epidemiologic cohorts of older adults, including the Tobago Health Study, MrOS, Mediators of Atherosclerosis of South Asians Living in America (MASALA), Multi-Ethnic Study of Atherosclerosis (MESA), The Southall and Brent Revisited (SABRE) study, Health ABC, and the UK Biobank. Notably, no published studies are available on myosteatosis among Native American adults who have the highest rates of diabetes in the US (U.S., 2023), highlighting an important gap in the literature.

Compared to older Caucasian men in MrOS (n = 1,105, mean age = 77 years), African Caribbean men in the Tobago Health Study (n = 518, mean age = 72 years) had significantly more calf IMAT (341.7 ± 10.9 mm^2^ vs. 419.2 ± 18.7 mm^2^; p < 0.001; Table 3) (Miljkovic et al., 2009a). Caucasian men were older, had a higher body mass index, and better scores on lifestyle measures, but results remained unchanged after adjusting for these lifestyle measures and health characteristics, and when matching for age and fat mass (Miljkovic et al., 2009a). These results contrast with other findings of myosteatosis by race. In a study of 338 adult African American, Asian, and White men and women, there was no difference in whole body IMAT by race at low levels of total adiposity, but African American people had more IMAT compared to White people at higher levels of total body adiposity (Table 3) (Gallagher et al., 2005). The differences in findings between these studies are unclear but could be due to the age of the study samples (all adults vs. middle-aged and older adults) or the anatomical location evaluated (calf, abdomen, whole body).

Compared to South Asians enrolled in the MASALA study (n = 2,627; mean age: 55 years), White men (not women), Chinese American men and women, African American men and women, and Latino men and women in MESA (n = 1,944; mean age: 62 years) had significantly less abdominal IMAT (Table 3), independent of demographic and lifestyle differences (Shah et al., 2016). South Asians had the highest amount of IMAT, followed by White, Latino, African American, and Chinese American. Notably, there was no formal statistical comparison with racial groups other than South Asians, so it is unclear if the difference in other races is significant. Results from the adults aged 40–69 years in the UK Biobank similarly showed that South Asians had significantly higher MRI-measured thigh muscle fat infiltration (defined as “the mean fat fraction in the ‘viable muscle tissue’“) compared to White European adults (age range: 40–69 years) across all BMI categories after adjusting for several demographic and lifestyle characteristics (mean difference: 0.69%, p < 0.001; Table 3) (Tejani et al., 2025). Thus, evidence suggests that South Asians have more myosteatosis compared to White adults.

Taking the race results further, some studies cross-sectionally compared IMAT and muscle density by race within sex. The Health ABC studies by Goodpaster et al. (2001) and Delmonico et al. (2009) mentioned previously found that Black women had greater IMAT and lower muscle density compared to White women (Table 4). Black women self-reported significantly more physical activity compared to White women (Delmonico et al., 2009). Thus, differences in physical activity likely do not explain racial differences in women since it would be expected that higher physical activity equates to less IMAT (Ramírez-Vélez et al., 2021). Black women, however, had significantly greater fat mass, fat free mass, body mass index, and total body mass (Delmonico et al., 2009), so differences in body size may account for observed race differences in women.

Both Health ABC studies also found that Black men had significantly greater IMAT compared to White men (p < 0.01), but no significant difference in muscle density (overall p = 0.17), except in the hamstrings (p = 0.008). As with women, Black men self-reported more physical activity compared to White men (though it was not statistically significant: p > 0.05). Black men had significantly less fat and fat free mass, but similar body mass index compared to White men (Delmonico et al., 2009), though the magnitude of difference in fat and fat free mass was small. Thus, neither physical activity nor body size likely accounts for the difference by race in men.

In the SABRE study (age range: 56–85 years), European men and women separately had significantly greater IMAT and muscle density compared to South Asian men and women, after adjusting for age and muscle area (Table 3) (Eastwood et al., 2014). These results would suggest that European men and women have greater IMAT, but less intra-muscular adipose tissue compared to South Asian men and women. However, all results were completely attenuated and not significant when adjusting for height and fat mass in both men and women, indicating that differences are attributed to body size. These results are in contrast with the study comparing MESA and MASALA; White men had significantly lower abdominal IMAT compared to South Asians (Shah et al., 2016). However, MESA and MASALA measured abdominal IMAT, while SABRE measured thigh IMAT, suggesting that IMAT may accumulate differently throughout the body by race with age.

Smaller, non-epidemiologic studies (sample size range: 15–73 per racial category) compared IMCL measured by MRI or MRS to evaluate differences by race among adults ≥18 years and were summarized by AlShehab et al. (2025). Briefly, eight studies examining IMCL by race found no difference between Black and White adults overall, but mixed results among women (AlShehab et al., 2025). South Asian men and American Indians showed slightly greater IMCL compared to White people (AlShehab et al., 2025). However, the quality of the findings was not described, nor were differences by age group in these studies, limiting our understanding of IMCL differences with age by race.

Together, results of myosteatosis with age by race suggest that the burden of myosteatosis in older adults is not shared equally by racial groups, particularly IMAT and intra-muscular adipose tissue depots for South Asian populations and for people with African ancestral origin. However, results are inconsistent, as one study found that Caucasian men had less IMAT compared to African Caribbean men, and the other showed that White older adults had more IMAT compared to African American older adults. However, there was no statistical comparison between White and African American people in the second study comparing MESA and MASALA. Additionally, Miljkovic et al. (2009a) compared calf IMAT in MrOS and the Tobago Health Study, while Shah et al. (2016) compared abdominal IMAT in MESA and MASALA. Therefore, it is not possible to directly combine the effects of these studies since we know that myosteatosis varies by muscle group (see “Importance of anatomical location”). Conversely, these observed differences by muscle group may also be due to physiological differences in accumulation; men of African ancestry may accumulate more IMAT in the thigh, while White men may accumulate more IMAT in the abdominal muscles. However, evidence from longitudinal studies is needed. Finally, none of these studies evaluated muscle density, so it is unclear how intra-muscular adipose tissue might differ by race. Nonetheless, these studies adjusted for several relevant covariates, including physical activity and lifestyle factors, hinting at a true racial difference with age that needs to be further evaluated.

The etiology of racial differences is unclear since relevant covariates were accounted for in the studies presented. Evidence from smaller studies in children suggests that there may be a potential genetic component or an influence of social, environmental, or structural determinants on myosteatosis accumulation that can be observed starting in adolescence (Lawrence et al., 2011; Liska et al., 2007; Maligie et al., 2012). Among adolescents with obesity (n = 55, mean age: 15 years), calf IMCL was significantly higher in Hispanic children compared to White children or African American children, despite similar age, weight, and gender by racial group (Liska et al., 2007). Hispanic and African American adolescents (n = 225, age range: 6–13 years) had significantly higher IMCL compared to non-Hispanic White adolescents (n = 197), after adjusting for body size (Maligie et al., 2012). This was similarly observed in a slightly older group of females (past puberty, ≤40 years; n = 57) with a body mass index in the overweight category, such that African American women had significantly greater extramyocellular lipid content compared to European Americans (Lawrence et al., 2011). In the Growing Up in Singapore Towards health Outcomes (GUSTO) birth cohort, IMCL in children aged 4.5 years was significantly higher in Indian children vs. Malay and Chinese children, after adjusting for maternal and offspring characteristics (Michael et al., 2020).

These findings demonstrate that racial differences in myosteatosis are identified even at a young age, suggesting either a potential genetic component, or a social, cultural, or environmental determinant that influences myosteatosis accumulation. There have been studies evaluating the heritability of myosteatosis, but none have evaluated the social or cultural influences that may contribute to racial and ethnic differences. Diet, access to care, and socioeconomic status are often not considered in analyses comparing myosteatosis by race, so these factors cannot be completely ruled out as a potential determinant of myosteatosis. Recent evidence suggests that environmental toxicants influence skeletal muscle health, specifically mitochondrial function (Clemens et al., 2026), which likely contributes to differences in racial and ethnic differences in myosteatosis. However, more evidence is needed on the social, cultural, and environmental influences of myosteatosis to tease out the contribution of each to myosteatosis accumulation differences by race.

Heritability results from the Tobago Family Health Study (Miljkovic et al., 2011) and analysis of genetic variants in mitochondrial enzymes in the Tobago Health Study (Miljkovic et al., 2009b) support a potential genetic link. Findings indicated that 33%–35% IMAT and intra-muscular adipose tissue amount are inherited (Miljkovic et al., 2011). C-reactive protein levels are correlated with intra-muscular adipose tissue (r = −0.10, p < 0.05) and C-reactive protein is about 40% (p < 0.001) heritable. Among adults who were homozygous for minor allele at G531 L and I66V, IMAT was 87% and 54% lower, respectively, suggesting a novel association between genetic variants in CPT1B and myosteatosis (Miljkovic et al., 2009b). Thus, there is evidence that myosteatosis is heritable, particularly in mitochondrial enzymes, among African Caribbean adults, which may explain the differences in children that persist into older adulthood. However, heritability data and other genetic studies of mitochondrial enzymes in other racial and ethnic groups are needed in epidemiologic studies, as are studies in middle-aged adults to see if this pattern persists throughout the lifespan.

Paradoxically, African American people tend to have more fast twitch (Type II) muscle fibers (Ama et al., 1986; Ceaser and Hunter, 2015) used for short bursts of activity, which contain lower levels of adipose tissue (Silva et al., 2010). However, this does not appear to translate to more favorable muscle composition since African American people tend to have more myosteatosis. Thus, differences in fiber type are likely not the explanation for increased myosteatosis with age by race. However, emerging evidence shows that there are differences in mitochondrial respiration by race (Jevtovic et al., 2022; Mucinski et al., 2025; Vezza and Víctor, 2025), further suggesting that genetic differences in mitochondrial DNA that result in differences in mitochondrial function and ultimately more muscle adiposity.

Taken together, these results suggest that myosteatosis is likely to differ by race, but results are inconsistent. These data are supported by studies in children and in genetic studies that suggest a lifetime difference in myosteatosis. However, heritability in other racial groups, studies of myosteatosis among middle-aged adults, and studies that evaluate the social and cultural determinants of myosteatosis are needed to describe racial differences across the entire lifespan and tease out the genetic versus social/cultural/environmental influences on myosteatosis. Additionally, more studies of mitochondrial differences and function by race are needed to determine if the significant signals identified are a cause for racial differences. Finally, Native American adults have the highest prevalence of diabetes, but no studies to date have published on muscle composition in this population, which further emphasizing the need for data in more diverse populations.

### 3.3 Importance of anatomical location, myosteatosis depot, and imaging modality

#### 3.3.1 Importance of anatomical location

Differences in myosteatosis across muscle groups likely exist. As mentioned above, findings from two FHS analyses reported age-related differences in muscle density varied by muscle group and sex. Trunk muscle density (T8 and L3) was 7.4–24.6 HU higher in younger (35–50 years) compared to older (75–87 years) adults (depending on the muscle) with greatest differences observed in paraspinal and abdominal muscles. Among a larger sample of FHS participants (n = 250; age range: 40–90 years), muscle density was 4%–17% lower in men and 6%–32% lower in women per 10-year age group (Johannesdottir et al., 2018), with greater age-related differences in the outer trunk muscles. A smaller study of community-dwelling older adults (n = 58, aged ≥65 years) similarly reported differences in muscle density and IMAT between abdominal, hip, and thigh muscles (Inacio et al., 2014), such that the gluteus maximus had the lowest muscle density and highest IMAT, and the rectus femoris had the highest muscle density and lowest IMAT. Thus, the gluteal muscles may be more susceptible to adipose tissue infiltration compared to the thigh or abdomen.

While published data on anatomical differences is limited, results to date suggest there are likely differences in age-related myosteatosis accumulation by muscle group. Further defining these anatomical differences in accumulation with age has relevant clinical impacts, as different locations influence risk of age-related outcomes. Higher myosteatosis in abdominal muscles, psoas, and hip muscles are associated with increased gait variability, decreased balance, increased risk of falls, and kyphosis progression (Correa-de-Araujo et al., 2020). Higher IMAT in the gluteal muscles may be associated with higher gait variability and poor balance (Addison et al., 2014c), while lower body myosteatosis is strongly associated with muscle strength and physical performance (Santanasto et al., 2023). Thus, one anatomical location likely does not represent myosteatosis throughout the body.

Of note, the conclusions drawn from this review used evidence from different muscle groups (e.g., calf, abdomen, thigh). We hypothesized that overall, myosteatosis increases with age regardless of the muscle group evaluated. However, the evidence presented in this section points to a potential difference in myosteatosis based on the muscle group measured. The overwhelming evidence points to an increase in myosteatosis with age, but the evidence from this section suggests that the rate may not be exactly the same throughout the body; myosteatosis increases in all muscle groups, but at a different rate or at different times depending on the muscle group. Future studies in this topic could further evaluate age-related differences in myosteatosis by muscle group among larger, epidemiologic cohorts over multiple measurements. Each of the studies reported was cross-sectional in design, limiting our understanding of the rate of accumulation throughout the body and the corresponding rate of disease progression. Thus, understanding the natural history of myosteatosis development may provide valuable insight into age-related declines in muscle composition at different locations and progression of age-related diseases.

#### 3.3.2 Importance of myosteatosis depot and imaging modality

Myosteatosis accumulation with age is not uniform across depots. In a cross-sectional study using a large sample of 850 men aged 50–95 years, Santanasto et al. (2023) reported that CT-measured thigh, calf, psoas, and paraspinal muscle density were all strongly correlated with one another (r range: 0.31–0.67, all p < 0.001) (Santanasto et al., 2023). However, observed correlations varied for IMAT: strong correlation between posterior muscles (r = 0.36, p < 0.001), weak correlation between leg muscles (r = 0.25, p < 0.001) and leg muscles with paraspinal muscles (calf and paraspinal: r = 0.12, p = 0.004; thigh and paraspinal: r = 0.27, p < 0.001), and no correlation with leg and psoas muscles (both p > 0.05).

The difference in strength of correlations between muscle density and IMAT suggests that myosteatosis accumulation by depot is site specific and possibly indicative of systemic influences on muscle density and local influences on IMAT. This difference in accumulation by depot is important to consider when selecting an imaging modality in epidemiologic studies since each myosteatosis depot is associated with different outcomes and each imaging modality measures a different depot. For example, CT measures IMAT and muscle density (a surrogate measure of intramuscular adipose tissue), while MRI can measure IMAT and IMCL. A more detailed comparison of imaging modalities can be found elsewhere (Correa-de-Araujo et al., 2020). As with the differences in anatomical location, while the accumulation may differ, this does not negate the evidence that each depot generally increases with age. However, a comparison of depots with age may be valuable, as one may indicate an earlier intervention before progression of myosteatosis in other depots. Of note, this review did not include studies that used ultrasound to measure myosteatosis. Ultrasound is a widely available and portable imaging methodology that could be used to measure myosteatosis of many different muscle groups throughout the body. However, there is no standardized protocol for ultrasound measures, and the myosteatosis measure varies depending on probe placement, pressure, and angle of incidence (Correa-de-Araujo et al., 2020). Additionally, ultrasound does not distinguish between intramuscular adipose tissue and IMAT. If more work is done to standardize ultrasound measures of myostetosis, this could be an easy and inexpensive method for measuring some depots of myosteatosis clinically.

## 4 Potential biological pathways of myosteatosis with age

The age-related increase in myosteatosis observed across sex and race has important clinical implications, as it is associated with mortality (Santanasto et al., 2017; Zhao et al., 2016) and long-term morbidity (Addison et al., 2014b), including metabolic (Miljkovic et al., 2021) and functional consequences (Dondero et al., 2024; Justice et al., 2018). Identifying biological mechanisms leading to age-related increases in myosteatosis may help elucidate potential therapeutic targets to slow the rate of accumulation and delay downstream clinical consequences. While the exact biological determinants of myosteatosis with age are unknown, there are several proposed pathways that may provide an opportunity for further exploration, including hormonal changes, cellular dysfunction, and lifestyle behaviors (Figure 2). This section broadly outlines some of most widely studied pathways, key regulators, and signaling mediators, though this review is not all-encompassing of every potential biological pathway.

### 4.1 Hormonal pathways of myosteatosis

On a hormonal level, both muscle and adipose tissue secrete signals known as myokines and adipokines, respectively. Leptin is an adipokine that helps maintain metabolic homeostasis through central and peripheral pathways (Carter et al., 2013). In a young, healthy adult, low leptin levels trigger the body to decrease energy intake and increase food intake, while high leptin levels trigger satiety (Flier and Maratos-Flier, 2017), increase insulin resistance and free fatty acid oxidation, and decrease lipolysis (Carter et al., 2013). Compared to younger adults of similar body weight, older adults have lower serum leptin levels, independent of age-related sex hormone differences (Isidori et al., 2000; Carter et al., 2013). The biological mechanisms of decreased leptin with age are unclear but could be due to changes in body adiposity with age (Considine et al., 1996), decreased expression of leptin receptors, and impaired leptin signaling (Fuentes et al., 2010; Guerra et al., 2014). Lower circulating levels of leptin in older adults may trigger increased energy intake (Zoico et al., 2013; Koteish and Diehl, 2001) and lower the body’s free fatty acid oxidation (Carter et al., 2013; Koteish and Diehl, 2001), both of which increase adipose tissue storage in the muscle.

Older adults often exhibit chronic low-grade inflammation even in the absence of overt disease. Elevated circulating inflammatory cytokines can impair muscle metabolism and lower lipid oxidation. As myosteatosis accumulates, IMAT specifically, further contributes to the elevated inflammatory environment through secretion of some inflammatory markers like inteleukin-6 (IL-6) and tumor necrosis factor-α (TNF-α). The presence of these inflammatory mediators disrupts skeletal muscle homeostasis and promotes further myostatosis accumulation, creating a cycle of myosteatosis progression (Dondero et al., 2024).

Another possible pathway is through impaired myokine secretion. Myostatin is a myokine that negatively regulates skeletal muscle growth and regeneration and has been evaluated as a therapeutic target to reduce skeletal muscle adipose tissue infiltration (Correa-de-Araujo et al., 2020; Dondero et al., 2024). Myostatin inhibits muscle protein synthesis, contributing to age-related loss of muscle mass and muscle quality. A phase two clinical trial of a myostatin monoclonal antibody demonstrated increased appendicular lean mass and improvements in functional power in older adults with a history of falls (Becker et al., 2015). This suggests that potentially inhibiting a pathway that negatively regulates muscle growth may improve myosteatosis and important muscle function outcomes. Additionally, myokine secretion is strongly impacted by exercise (Ha et al., 2025) and the increasingly sedentary lifestyle of older adults may impair myokine secretion, ultimately influencing fat mass accumulation (Ha et al., 2025) and muscle mass maintenance (Lee and June 2019). However, this pathway may be bidirectional and is an emerging area of research, so it is difficult to fully tease out its role in myosteatosis. Thus, the impaired adipokine and myokine secretion, as well as increased inflammation with age, likely contributes to increased skeletal muscle adiposity with age.

### 4.2 Cellular origins of myosteatosis

There are also some potential cellular origins of myosteatosis that may be a direct result of aging, including alterations to satellite cell differentiation or progenitor cell function (Zhang et al., 2023). Myoblasts are derived from satellite cells that typically differentiate into muscle cells but also can differentiate into adipocytes under certain circumstances (Zhang et al., 2023). Myoblast differentiation into adipocytes (instead of muscle cells) commonly occurs with age, as intrinsic and extrinsic damage or alterations can hinder satellite cell differentiation and regenerative capacity (Parker, 2015). Subcutaneous adipose tissue releases adipocyte progenitor cells that promote IMAT, particularly in the presence of caloric overload (Girousse et al., 2019). Mesenchymal progenitor cells can differentiate into muscle, adipose, or bone cells. Mesenchymal cells that are derived from muscle are known as fibro/adipogenic progenitors (FAPs) and are responsible for muscle tissue regeneration and maintaining muscle size (Parker and Hamrick, 2021). The role of FAPs are regulated by a complex network of signals (Parker and Hamrick, 2021), including peroxisome proliferator-activated receptor gamma (PPARγ), a key regulator of adipogenesis, and transforming growth factor-beta (TGF-β), which influences fibrotic and adipogenic remodeling (Flores-Opazo et al., 2024). In response to muscle injury or periods of low grade inflammation (e.g., aging) (Dondero et al., 2024), dysregulation of FAPs results in the progenitor cells differentiating into adipocytes that are deposited within and between the muscle, which results in increased myosteatosis. However, the interrelationship between FAPs and myosteatosis is so complex and largely unknown, so more research on regulators of FAPs would be helpful for further understanding this complex network.

Mitochondrial function and content also change with age (Boengler et al., 2017) and injury (Gumucio et al., 2019). Physical activity upregulates peroxisome proliferator-activated receptor gamma coactivator-1 alpha (PGC-1α), which signals fatty acid oxidation (Iijima et al., 2025). With the increasingly sedentary lifestyle of older adults, PGC-1α is reduced, impacting mitochondrial fatty acid oxidative capacity. While adipose tissue tends to have fewer mitochondria compared to other tissues like muscle and heart, the declining mitochondrial function with age can greatly impact adipose tissue metabolism and differentiation (Boengler et al., 2017), leading to accumulation of adipocytes within the muscle. Thus, a few cellular origins may be contributing to myosteatosis accumulation, including mitochondrial dysfunction, impaired satellite cell differentiation, and impaired progenitor cell function.

### 4.3 Lifestyle factors contributing to myosteatosis

From a lifestyle perspective, changes in diet and exercise with age likely contribute to higher amounts of myosteatosis. Diet quality among US adults decreased between 2001 and 2018, with poor quality diet remaining consistently low in older adults (Long et al., 2022). Similarly, physical activity levels decline with age among older adults (Keadle et al., 2016). The increasingly poor quality diet and sedentary lifestyle with age seemingly corresponds to the increase in myosteatosis with age. There are randomized trials evaluating the impact of diet and exercise interventions on myosteatosis, but sample sizes are small and not generalizable to the entire older adult population. Poor diet quality, specifically high fat and high calorie diet, is associated with greater myosteatosis in adults in randomized trials (Ahmed et al., 2018). However, these dietary interventions had small sample sizes (range: 6–66) and had an age range or average age <65 years, so the magnitude of association may not be the same in older adults. Intervention studies in adults also show that myosteatosis may be responsive to diet and exercise interventions if the intervention leads to weight loss (Prokopidis et al., 2025; Addison et al., 2014b), with effects of weight loss by exercise being superior to diet (Addison et al., 2014a). Similar to dietary intervention studies, there are few physical activity intervention studies in older adults specifically (Goodpaster et al., 2008; Santanasto et al., 2011; Taaffe et al., 2009; Marcus et al., 2010) and published data are from small sample sizes (n = 13–42). A recent meta-analysis of randomized trials comparing an exercise intervention to controls in adults aged ≥18 years found that exercise significantly decreased IMAT and increased muscle density (Ramírez-Vélez et al., 2021). However, the age range of studies was anywhere from 28 to 77 years, sample sizes were small, and there was significant heterogeneity in the summary estimate. Thus, evidence exists that changes to lifestyle may improve myosteatosis levels, but it is unclear if these results translate to the larger population of older adults.

Many of the studies reported in this review did not control for diet, so diet likely contributes some to the age-related increase in myosteatosis observed in the reported studies. However, many studies adjusted for physical activity levels, suggesting that myosteatosis continues to increase with age regardless of activity levels. Many measures of physical activity were self-reported and not specific to older adults (Anderson et al., 2013), were validated in women only (Shah et al., 2016), or included only one exercise modality (Miljkovic et al., 2009a), so results may not truly reflect physical activity levels. Even among older adults who self-reported being lifelong exercises, IMAT increased with age, though to a lesser extent than older adults who did not report being a lifelong exerciser (Chambers et al., 2020), further supporting the age-related increase in myosteatosis.

Taken together, improving diet quality and increasing physical activity may decrease myosteatosis, but current evidence is not sufficient to suggest that lifestyle changes can completely prevent accumulation with age. Importantly, the available evidence from randomized trials is limited to small sample sizes of adults who are likely healthier than the general older adult population since they were willing and able to participate in a physical activity intervention. Larger randomized trials of diet and physical activity interventions are needed in older adults to determine if lifestyle slows the progression of myosteatosis at scale. Additionally, observational studies need to adjust for diet and adjust for physical activity measures that are more comprehensive (e.g., device measures or activity type) and validated in older adults specifically to further tease out the effects of lifestyle on myosteatosis accumulation.

## 5 Clinical consequences of myosteatosis accumulation

The age-related increase my myosteatosis has important clinical implications (Figure 2), as it is associated with several systems, including the musculoskeletal system, cardiometabolic disease, cognition, and survival. Given its close proximity to skeletal muscle, evidence suggests that higher amounts of myosteatosis are associated with progressive muscle weakness (Delmonico et al., 2009; Roe et al., 2025) and mobility impairments (Santanasto et al., 2019; Visser et al., 2005) by impairing the local muscle environment (Yoshida et al., 2012), increasing proinflammatory cytokines (Addison et al., 2014a), and impaired myokine secretion (Leal et al., 2018). Notably, there are no studies of myosteatosis and its association with skeletal muscle power or powerpenia. The number of type II muscle fibers declines with age, contributing to declines in skeletal muscle power with age. Muscle power is a critical component of skeletal muscle function that is responsible for dynamic balance, joint stabilization, and protection against falls and fall injuries. Thus, this presents an important gap in the literature. To more comprehensively understand the impact of myosteatosis on skeletal muscle function, future work could evaluate the association between myosteatosis and muscle power.

In older adults, higher myosteatosis is associated with lower compartmental bone mineral density (Chan et al., 2018) and higher fracture risk, independent of bone mineral density (Sheu et al., 2013). The association with bone may be due to the overall decline in muscle composition and the underlying metabolic changes that are strongly associated with myosteatosis in epidemiologic studies (Goodpaster et al., 2023; Miljkovic et al., 2021). Adipose tissue is an endocrine organ that directly impacts insulin sensitivity, likely through increased inflammation, fibrosis, and increased local free fatty acid concentration (Sachs et al., 2019). Recent publications found that greater myosteatosis is associated with lower processing speed, an early indicator of cognitive impairment (Acevedo-Fontánez et al., 2023; McNeish et al., 2025; Acevedo-Fontánez et al., 2025), though the pathway through which this association occurs is being explored. Thus, the consequences of increased myosteatosis are not immaterial and can contribute to several detrimental downstream consequences in several physiologic systems. Given the evidence presented in this review of the age-related increase in myosteatosis, it is important to continue to understand this adipose tissue depot, as it could be an easily-measured marker that is indicative of future health events. However, cutpoints for myosteatosis have not been established, making it difficult for clinicians to understand what a “healthy” level of myosteatosis is and what is considered a “normal” rate of accumulation. Future research to establish cutpoints and to make myosteatosis clinically accessible would greatly improve its clinical utility.

## 6 Conclusion

We summarized age-related changes in myosteatosis from population-based epidemiologic cohort studies and evidence supports myosteatosis as a potential marker of aging, independent of weight change, body size, activity level, or chronic disease burden. Myosteatosis is an important contributor to functional decline, metabolic consequences, and other adverse outcomes (Figure 2), and the epidemiologic evidence from this review highlights its primary role as a potential marker of aging. Prior reviews provided brief discussions on changes in myosteatosis with age (Addison et al., 2014b; Correa-de-Araujo et al., 2020; Tordjman et al., 2025; Zhang et al., 2025; Yang et al., 2026). Findings from the present review more extensively support the prior discussions and further suggest that myosteatosis does not accumulate uniformly with age across sex, race, or anatomical location. Specifically, older women tend to have more intra-muscular adipose tissue compared to older men, and South Asians tend to have the most IMAT, followed by African American people and White people. Importantly, this review includes more recent longitudinal studies, further supporting the notion that myosteatosis may increase with age. Exact biological mechanisms driving age-related increases in myosteatosis are unknown, but several cellular, genetic, hormonal, and lifestyle factors likely contribute. Studies in children and older adults comparing myosteatosis by race show that differences are observed as early as adolescence, potentially suggesting heritability, but it is largely unknown if or how this might vary in mid-life. While physical activity or diet may slow the increase in myosteatosis with age, there is currently not enough evidence to show that it stops the age-related accumulation.

Several questions about myosteatosis accumulation with age remain unanswered, particularly surrounding the influence of subclinical disease that may lead to weight loss or increased inflammation. The older adult population is heterogeneous, so there is a chance that many older adults included in this review had some level of subclinical disease. While this review included many large-scale, population-based studies of older adults that adjusted for chronic conditions, it is challenging to completely rule out the role of weight loss, inflammation, and subclinical pathology. The literature would benefit from evaluation of myosteatosis in healthy, long-lived individuals without any subclinical disease to understand if myosteatosis accumulates in even the healthiest older adults. Additionally, cohort studies with measures of subclinical disease and data on intentionality of weight loss may be beneficial to further teasing out the role of aging on myosteatosis.

This review is strengthened by our inclusion of several different population-based epidemiologic cohorts with larger sample sizes that allow for greater generalizability of findings to older adults and are powered to detect meaningful differences in myosteatosis by age. Additionally, many of these studies adjusted for covariates that could impact myosteatosis with age, including chronic disease, physical activity, and weight change.

However, there are several limitations to the literature, including lack of longitudinal data that show the natural history of myosteatosis throughout adulthood and into older adulthood. There is also a lack of longitudinal data that includes biological markers to understand how potential pathways leading to age-related increases in myosteatosis change over time at a population level. Given the small number of studies with longitudinal data, there is potential for reverse causality, such that increasing chronic disease prevalence with age may in turn increase myosteatosis. Epidemiologic cohort studies are observational in nature, meaning there is a risk of unmeasured confounding that may bias the data away from the null. Thus, the possibility remains that these findings are a result of unmeasured subclinical pathology or inflammation that accompany aging, but the consistency of findings across studies suggests that this is less likely the reason for the observed associations in each of the studies. In older adults specifically, weight loss can be controversial depending on if the weight loss is intentional or unintentional, but studies that evaluated myosteatosis by weight change were not able to distinguish intentionality. Unintentional weight loss often signals underlying disease processes, which would potentially be accounted for when studies controlled for disease status, but it could also be indicative of subclinical disease that has yet to be identified and was therefore, not included in analyses. Physical activity was broadly controlled for using self-reported questionnaires, but device measures of physical activity and evaluation of activity type likely impact ectopic fat storage differently. Much of the literature did not include diet as a covariate in models, so we cannot rule out the role of diet on myosteatosis with age. Finally, anatomical site and imaging methods used to evaluate myosteatosis varied greatly, which introduces inherent variability in findings. As others have noted (Zhang et al., 2025; Ahn et al., 2021; Yang et al., 2026), the lack of standardized imaging methods limits our ability to set clinical cutpoints or population norms for older adults to specifically quantify elevated risk. Additionally, we did not include any studies that utilized ultrasound imaging techniques, as this method is not common in epidemiologic studies, there is a lack of standardization for protocols, and there is inherent variability in the resulting measures (Zhang et al., 2025). However, given the clinical accessibility, portability, and ease of use, ultrasound could be used in the future as a more widely applicable measurement method. Though the variability can high, there is potential to measure muscle-specific myosteatosis depots that are important to an individual’s risk profile.

In conclusion, evidence from several population-based epidemiologic studies suggests that myosteatosis is a potential independent marker of aging among the general population of older adults. These findings provide another possible avenue for understanding muscle function declines with age.
